# Cardiovascular Responses during Head-Down Crooked Kneeling Position Assumed in Muslim Prayers

**Published:** 2013-06

**Authors:** Adamu Ahmad Rufa’i, Hadeezah Hamu Aliyu, Adetoyeje Yunoos Oyeyemi, Adewale Lukman Oyeyemi

**Affiliations:** 1 Department of Physiotherapy, University of Maiduguri, Maiduguri, Nigeria;; 2 Department of Physiotherapy, Aminu Kano Teaching Hospital, Kano, Nigeria

**Keywords:** Cardiovascular system, Head-down, Muslim, Prayers

## Abstract

**Background**: Movement dysfunction may be expressed in terms of symptoms experienced in non-physiological postures, and head-down crooked kneeling (HDCK) is a posture frequently assumed by Muslims during prayer activities. The purpose of this study was to investigate the cardiovascular responses in the HDCK posture.

**Methods: **Seventy healthy volunteers, comprising 35 males and 35 females, participated in the study. Cardiovascular parameters of blood pressure and pulse rate of the participants were measured in rested sitting position and then at one and three minutes into the HDCK posture. Two-way ANOVA was used to determine the differences between cardiovascular responses at rest and in the HDCK posture, and the Student *t* test was utilized to determine gender difference in cardiovascular responses at rest and at one and three minutes into the HDCK posture.

**Results: **The study showed a significant decrease in systolic and diastolic blood pressures at one minute into the HDCK posture and an increase in pulse rate at one and three minutes into the HDCK posture, as compared to the resting values. Rate pressure product also rose at one minute into the HDCK posture, whereas pulse pressure increased at one and three minutes into the HDCK posture, as compared with the resting values. However, no significant change was observed in the mean arterial pressure values.

**Conclusion: **The findings from this study suggest that no adverse cardiovascular event can be expected to occur for the normal duration of this posture during Muslim prayer activities.

## Introduction

Cardiovascular variables such as blood pressure and pulse rate are influenced by a wide range of factors such as emotion, lifestyle, and physical activities including changes in posture.^1^ Normal physiological postures, i.e. fundamental starting positions, include supine lying, side lying, sitting, and standing, while tilting and inversion are derived non-physiological postures. These two postures (especially inversion) are unusual in daily life activities. However, in physiotherapy procedures, tilting position combined with deep breathing exercise, percussion, and vibration helps remove excess secretions in pulmonary conditions.^2^^-^^5^ Moreover, acrobats, children, and military personnel sometimes assume momentary tilted or inverted positions during play and maneuvers.

Several studies have been conducted to investigate the physiological responses during non-physiological postures and activities. Head-down position during long periods of sitting decreases heart rate, increases ejection duration, and causes shift in the myocardial oxygen supply/demand ratio.^6^ On assumption of a passive head-down tilt, blood is shifted towards the upper thorax and head, consequently, the forearm blood flow and arterial blood pressure increase and heart rate decreases.^7^^,^^8^ Mengesha,^9^ and Balogun et al.^10^ reported that systolic and diastolic blood pressures as well as mean arterial blood pressure (MAP) and pulse pressure (PP) were not affected by aerobic training and head-down inversion, but pulse rate and rate pressure product (RPP) during head-down inversion were significantly reduced.

The assessment of cardiovascular responses during orthostatic stress resulting from varying postures may provide important information on the regulation and control of blood pressure across different races and genders.^11^^-^^13^ From the extant literature, the majority of studies on cardiovascular responses during non-physiological postures have been conducted in the industrialized nations,^8^^,^^14^^,^^15^ and the few relevant studies from the developing countries were conducted over two decades ago.^10^ In physiotherapy clinics, patients usually present with problems such as limitation in daily life activities, which may include limitations in assuming postures, e.g. during various prayer positions. 

In Muslim prayer activities, postures assumed encompass standing, bowing or Ruku’u, and head-down crooked kneeling (HDCK) or Sujood (prostration), and sitting in that order. Head-down inversion in Sujood is similar to poses used in hatha yoga, which is a practice that focuses on relaxation, body awareness, and meditation.^16^ There are a total of 34 prostrations in the usual five daily obligatory prayers, during which each HDCK posture lasts for approximately 10 to 15 seconds. In the unusual or periodic non-obligatory prayers observed during the Ramadan fasting period or Taraweeh and the late night prayer or Tahajjud, prostration usually lasts for up to 2 minutes or even longer. 

None of the previous studies has explored cardiovascular responses during the HDCK posture, assumed during Muslim prayer activities. The objective of this study was to investigate the cardiovascular responses of healthy subjects during prostrations in Muslim prayers. We hypothesized that there would be no significant difference in cardiovascular parameters at rest and during Sujood and that there would be no significant gender difference in cardiovascular parameters at different time points during Sujood.

## Methods

*Participants*

Seventy healthy young students of the University of Maiduguri and staff of the University of Maiduguri Teaching Hospital (35 males and 35 females) participated in the study. Subjects with a known history of chronic headache, glaucoma, hypertension, or other related heart diseases were excluded and informed consent was obtained from the participants before data collection.

*Instruments*

The following instruments were used for the collection of data in the study: digital electronic device to monitor blood pressure and pulse rate (Beurer D-89077 Germany), bathroom scale, Standiometer, stop watch, table mat, and data collection instruments such as data forms and pens. 

The study was conducted at the gymnasium of the Department of Physiotherapy, University of Maiduguri Teaching Hospital.

*Study Procedure*

A repeated measurement design was utilized for this study. The participants were interviewed for medical history of any cardiovascular ailment, and those who met the inclusion criteria were given a consent form to sign. A pilot study was carried out prior to the main study, which revealed that the participants started experiencing discomfort at three minutes into Sujood.

The participants were advised not to eat kola nuts (kola nitida) and avoid drinking caffeine-containing beverages 24 hours before the commencement of the study. All the measurements were taken in the morning because of the hot weather condition during the study period. On arrival at the lab, the participants received explanation about the protocol of the study and their heights and weights were measured using a Standiometer and a bathroom scale, respectively. Afterward, the participants assumed sitting position.

Five minutes after the assumption of sitting position, resting period blood pressure and pulse rate were measured. The measurements were repeated at the seventh minute and the average of the two readings was recorded. Subsequently, the participants assumed prostration and stayed in that position for up to three minutes, during which time, blood pressure and heart rate were monitored at one and three minutes into prostration. The participants then returned into sitting position and their blood pressure and pulse rate were monitored for a duration of five minutes before discharge.

For each participant, MAP, RPP, and PP, as dependent variables, were computed. MAP is an important predictor of cardiac output,^17^ RPP is a valid predictor of myocardial oxygen demand, and PP is a good predictor of stroke volume.^10^

The ethical approval of the Ethics Committee of the University of Maiduguri Teaching Hospital was obtained before the commencement of this study.

*Data Analyses*

Data analysis was performed using Statistical Package for Social Sciences (SPSS version 16.0). Descriptive statistics of mean and standard deviation were drawn upon to describe the participants’ physical characteristics and to describe the cardiovascular responses at rest and at different time points during the HDCK position, i.e. at one and three minutes into prostration.

Inferential statistic of the independent *t*-test was employed to determine differences in the physical characteristics between the male and female participants, and the analysis of variance (two-way ANOVA) was utilized to determine differences in the cardiovascular responses between the male and female participants at rest and during the HDCK position. Least Significance Difference (LSD) was used as a post-hoc test to probe significant main effects, and a significance level of 0.05 was adopted in the study.

## Results

The mean age of the participants was 27.73±6.64 years. The mean height and weight of the male participants were significantly higher than those of the females, as is shown in table 1. Table 2 shows the gender differences in the participants’ cardiovascular responses to the HDCK position. There was no significant difference in the baseline diastolic blood pressure (P=0.14), RPP (P=0.20), and PP (P=0.38) between the males and females. The male participants had significantly higher baseline systolic blood pressure (P<0.001) and MAP (P<0.002) than the females, while the females had significantly higher baseline pulse rate (P<0.001) than the males. Furthermore, the systolic pressure and MAP were significantly higher (P<0.001) among the males than the females at one and three minutes into the HDCK position. Pulse rate was significantly higher among the female than the male participants at one and three minutes into the HDCK position (P<0.001). 

**Table 1:** Physical characteristics of the participants and differences by gender

**Variables**	**Male**	**Female**	**Combined mean(SD)** **n=70**	**t **	**P value**
Age (years)	29.3±7.5	26.2±5.3	27.73±6.64	2.00	0.49
Height (cm)	172.3±9.6	162.2±5.8	167.7±9.37	5.30	0.00*
Weight (kg)	70.4±13.2	59.2±10.4	64.83±13.06	4.00	0.00*
BMI (Kg/m^2^)	23.65±3.34	22.51±3.77	23.07±3.58	1.34	0.19

*Indicates significant difference at P<0.05; BMI: Body Mass Index

**Table 2:** Cardiovascular responses and differences by gender at rest and at one and three minutes into Sujood

**Variables**	**Gender**		**P value**
	**Male mean (SD)**	**Female mean (SD)**	
SBP (mm Hg)			
Rest	122.66±10.66	113.63±09.91	<0.001*
First min	120.17±09.62	109.03±11.50	<0.001*
Third min	122.14±12.45	110.31±10.22	<0.001*
DBP (mm Hg)			
Rest	82.89±10.49	78.74±12.70	0.141
First min	78.50±18.96	72.46±12.04	0.020
Third min	79.09±09.05	75.09±11.96	0.119
PR (bpm)			
Rest	69.86±08.23	78.71±10.70	<0.001*
First min	70.20±09.80	79.09±10.89	<0.001*
Third min	72.37±10.33	80.29±12.07	<0.001*
RPP			
Rest	8551.86±1078.75	8933.34±1390.45	0.20
First min	8580.14±1347.51	8615.43±1347.50	0.48
Third min	8836.51±1568.75	8851.17±1529.12	0.96
PP			
Rest	39.77±9.29	34.89±10.03	0.38
First min	41.66±9.28	36.57±08.77	0.21
Third min	43.06±10.94	35.23±09.13	<0.001*
MAP			
Rest	96.14±9.38	90.37±10.86	0.020*
First min	92.40±8.08	84.65±11.12	0.001*
Third min	93.44±8.92	86.83±10.56	0.006*

*Indicates significant difference at P<0.05; SD: standard deviation; SBP: systolic blood pressure; DBP: diastolic blood pressure; PR: pulse rate; MAP: mean arterial pressure; RPP: rate pressure product; PP: pulse pressure; bpm: beats per minute

Table 3 illustrates the effect of time during the HDCK position and gender on the cardiovascular responses of the participants. Significant differences were found in the time frames spent in the HDCK position for all the cardiovascular parameters, except for PP (F=2.02, P=0.13). The varying effect of time in the HDCK position was not significantly different between the male and female participants for all the cardiovascular parameters.

**Table 3:** Effect of time and group on cardiovascular responses of the participants

**Variables**	** MS**	**df**	**f**	**P value**
SBP (mm Hg)				
Time	220.13	2	8.32	<0.001*
Time x Group	37.28	2	1.41	0.25
DBP (mm Hg)				
Time	523.32	2	13.42	<0.001*
Time x Group	23.05	2	0.59	0.50
PR (bpm)				
Time	83.30	2	4.01	0.02
Time x Group	5.35	2	0.26	0.77
RPP				
Time	2215963.12	2	4.03	0.02*
Time x Group	596792.60	2	1.08	0.34
PP				
Time	75.61	2	2.20	0.13
Time x Group	47.31	2	21.26	0.28
MAP				
Time	405.45	2	15.30	<0.001*
Time x Group	17.30	2	0.65	0.52

*Indicates significant difference at P<0.05; SBP: systolic blood pressure; DBP: diastolic blood pressure; PR: pulse rate; RPP: rate pressure product; PP: pulse pressure; MAP: mean arterial pressure; MS: mean of squares; DF: degree of freedom; bpm: beats per minute

Post-hoc analysis revealed that the significant differences (P<0.001) in systolic blood pressure were between baseline (118.14±10.90) and at one minute into the HDCK position (114.60±11.93) and between baseline (118.14±10.90) and at three minutes into the HDCK position (116.23±12.78). Diastolic blood pressure was significantly reduced (P<0.001) at one minute into the HDCK position (74.49±10.9) from the baseline value (80.81±11.75), and it also significantly decreased (P=0.005) at three minutes into the HDCK position (77.09±10.72) from the baseline value. For pulse rate, responses were significantly elevated (P=0.014) at three minutes into the HDCK position (76.33±11.84) from the baseline value (72.29±10.27), and also after the third minute (76.33±11.84) from the first minute (74.64±11.22) into the HCDK position. MAP was significantly reduced (P<0.001) at one minute into the HCDK position (88.52±10.41) from the baseline value (93.26±10.48), and was also significantly reduced (P=0.021) at three minutes into the HDCK position (90.13±10.26) from the baseline value (93.26±10.48). A significant difference (P=0.003) was found only between the first and third minutes into the HDCK position for RPP, with the value significantly higher at three minutes into the HDCK position (8843±1537.81) than at one minute into the position (8497.79±1402.20) (not shown in the table).

## Discussion

The aim of this study was to investigate the cardiovascular responses of healthy subjects during the HDCK position, which is assumed in Muslim prayers. We hypothesized that there would be no significant difference between the cardiovascular parameters of the male and female participants at different time points during Sujood. The main finding was that systolic and diastolic blood pressures were significantly reduced in both male and female participants following Sujood, in comparison with the baseline values, but these reductions were not sustained through the third minute into the position. This finding is relatively concordant with that of the the study of White and Mawdsley,^18^ which reported a significant decrease in systolic and diastolic blood pressure response among subjects in side-lying, 10° head-down tilt. The finding is, however, inconsistent with that of LeMarr et al.^19^ which reported a consistent increase in systolic blood pressure during a three-minute inversion. Also, contrary to the Klatz et al.^20^ and Ballantyne,^21^ studies, which showed an average increase of 20 mm Hg for systolic blood pressure during inversion, our findings revealed a lower increase of 7 mm Hg in systolic blood pressure at one minute into the HDCK position. 

The inconsistency with respect to systolic and diastolic blood pressures between our findings and those of some previous studies^19^^-^^21^ may be attributed to the different position and duration of tilting and inversion used. In contrast to the present study, inversion and tilting in all the previous studies were assumed for longer durations. It is also possible that the attenuation response in blood pressure to inversion in this study was due to the adaptation of baroreceptors to head-down fatigue in the participants insofar as the majority of them were Muslims, who frequently adopt this position during their daily prayers.

Contrary to the finding of Klatz et al.^20^ who found no increase in pulse rate during more than 90° head-down inversion among healthy young subjects, a significant increase in pulse rate was found at three minutes into inversion in the present study. The increase in heart rate response from the resting value, found in the present study, was very much expected because anxiety, albeit subtle, always occurs during unusual positions and can trigger a sympathetic pressure response.^22^ According to the law of hydrostatics, circulatory pressure differences are produced by the three phenomena of gravity force, blood density, and the vertical distance between the two points being measured. Also, Starling’s law stipulates that both cardiac output and systemic blood pressure are expected to rise following changes in postures. This situation would probably influence pulse rate in either way. A decrease may result from blood distribution, which would influence the baroreceptors to cause vagal stimulation and augment response, hence giving rise to the reflex vasodilatation of the peripheral bed.^13^ An increase may also result to ensure continued evacuation of blood from the dependent region in the unusual posture, especially in less efficient circulation as in sedentary participants. This could be more likely since the veins and muscles of the upper part of the body are not specially adapted as the veins and muscles of the legs to aid venous return to the heart.

Our results, documenting a rise in MAP and RPP between the first minute and third minute into the HDCK position and no change in PP throughout the maneuver, are consistent with those of Balogun et al.^10^ These changes in MAP and RPP, which were not observed between the baseline values and at three minutes into the position, suggest an initial reduction before an upward trend in these values as the subjects assumed the HDCK position. Consistent with the study of Balogun et al.^10^ our findings show a decrease in MAP at one minute into prostration (as compared to the resting value), an increase at three minutes into prostration (as compared to the first minute value), and a decrease in MAP at three minutes into prostration (as compared with the resting value) (P>0.05).

Contrary to the expectation that cardiovascular responses following Sujood would be different between males and females, it is worth noting that the changes in the cardiovascular parameters at different time points during the HDCK position were not significantly different between the male and female participants in this study. This finding in our sample seems to confirm the conclusions that blood pressure responses to varying positions are comparable irrespective of the gender and race of the participants.^12^ Perhaps gender is not a personal individual factor that can influence or determine differences in cardiovascular responses following varying postures. 

There are some limitations to this study, which should be noted when interpreting the findings. First, the external validity of the study may be low because the participants were recruited through a convenience sampling technique and were limited to people with a potentially higher educational status (university students and staff of a teaching hospital). Second, the measurement was not performed at a similar time for all the participants, and it is possible for cardiovascular responses to inversion to vary at different time points during the day when the measurements are taken. Also, the environmental temperature during the procedure was not measured, which may preclude the generalization of our findings to different seasons in Maiduguri, Nigeria. 

Despite these limitations, however, the findings from the present study can be used as a basis for further exploratory studies on the cardiovascular responses to inversion during Muslim prayers, and can also elucidate on the body of knowledge on the effects of postural changes on cardiovascular responses in the general population. 

## Conclusion

This study revealed a significant decrease in systolic and diastolic blood pressures and an increase in pulse rate at different time points during Sujood. Also, our findings showed no significant gender difference in the effect of Sujood on cardiovascular parameters at different points in time. These findings indicate that the blood pressure of the healthy subjects was not elevated within the first three minutes into Sujood and suggest that there may be no adverse effect or danger attributable to this prayer position. 

The present study is confirmation that Sujood is safe and no adverse cardiovascular effect can be expected to arise from it in the usual obligatory Muslim prayers or even longer durations for which the unusual or periodic non-obligatory prayers are held.
